# GenePainter: a fast tool for aligning gene structures of eukaryotic protein families, visualizing the alignments and mapping gene structures onto protein structures

**DOI:** 10.1186/1471-2105-14-77

**Published:** 2013-03-04

**Authors:** Björn Hammesfahr, Florian Odronitz, Stefanie Mühlhausen, Stephan Waack, Martin Kollmar

**Affiliations:** 1 Department of NMR-based Structural Biology, Max-Planck-Institute for Biophysical Chemistry, Am Fassberg 11, Göttingen, 37077, Germany; 2 Institute of Computer Science, University of Göttingen, Goldschmidtstr. 7, Göttingen, 37077, Germany

**Keywords:** Exon, Intron, Gene structure, Evolution

## Abstract

**Background:**

All sequenced eukaryotic genomes have been shown to possess at least a few introns. This includes those unicellular organisms, which were previously suspected to be intron-less. Therefore, gene splicing must have been present at least in the last common ancestor of the eukaryotes. To explain the evolution of introns, basically two mutually exclusive concepts have been developed. The introns-early hypothesis says that already the very first protein-coding genes contained introns while the introns-late concept asserts that eukaryotic genes gained introns only after the emergence of the eukaryotic lineage. A very important aspect in this respect is the conservation of intron positions within homologous genes of different taxa.

**Results:**

GenePainter is a standalone application for mapping gene structure information onto protein multiple sequence alignments. Based on the multiple sequence alignments the gene structures are aligned down to single nucleotides. GenePainter accounts for variable lengths in exons and introns, respects split codons at intron junctions and is able to handle sequencing and assembly errors, which are possible reasons for frame-shifts in exons and gaps in genome assemblies. Thus, even gene structures of considerably divergent proteins can properly be compared, as it is needed in phylogenetic analyses. Conserved intron positions can also be mapped to user-provided protein structures. For their visualization GenePainter provides scripts for the molecular graphics system PyMol.

**Conclusions:**

GenePainter is a tool to analyse gene structure conservation providing various visualization options. A stable version of GenePainter for all operating systems as well as documentation and example data are available at http://www.motorprotein.de/genepainter.html.

## Background

All eukaryotic genomes that have been sequenced so far have been shown to possess at least a few introns including the unicellular organisms that were previously suspected to be intron-less [1,2]. These data has fuelled the lively debate between the introns-early and introns-late concepts that is ongoing since the discovery of splicing [3]. The introns-early hypothesis says that already the very first protein-coding genes contained introns while the introns-late concept asserts that eukaryotic genes gained introns only after the emergence of the eukaryotic lineage. Support for either of the concepts has been revealed by modelling the rates of intron gain and loss in eukaryotic genomes [4], by analysing the conservation of intron positions of example genes from a selection of genomes [5], or by population-genetic considerations [6]. Intron position conservation has also been used to improve gene predictions [7] and multiple protein sequence alignments [8], and to reconstruct ancient genes [9].

A few software packages are available for the analysis of the conservation of intron positions. Exalign is a software using only gene structure information to reveal intron conservation [10]. Exalign uses gene structures from RefSeq gene annotations and from user definitions and calculates an alignment solely based on exon lengths and reading frames. Accordingly, Exalign fails if exon lengths between genes do not match. This is usually the case if genes encode less conserved proteins (e.g. differing in the lengths of surface loop regions) or if genes from more divergent species, which have been subject to complex intron loss and gain events, are compared. To overcome these limitations, it would be beneficial to use the information contained in protein sequence alignments. Tools that combine protein multiple sequence alignment (MSA) data and gene structures are CIDA/CIWOG [11], GECA [12], Malin [13], and scripts developed for large-scale analyses [14,15]. CIDA/CIWOG comes with a web interface coupled to a database thus providing a barrier for installation while Malin requires a species phylogeny as starting point. GECA builds on the CIWOG output and provides the visualization of sequence similarity between subsequent genes. However, multiple sequence alignments are automatically generated and there is no option to use manually improved own alignments. In addition, sequence similarity is only computed for subsequent genes, which is inappropriate for large data sets. XdomView is the only software combining protein structures with intron and domain positions [16]. In XdomView the user specifies a PDB-ID and domain definitions from SCOP, CATH, DALI, 3DEE, and MMDB are subsequently mapped to the specified structure. In addition, the protein sequence from the PDB file is used to identify eukaryotic homologs in the ExInt database to map intron positions and phase. However, XdomView is strongly limited by accepting only PDB codes as input and the reference databases are far out of date (PDB of June 2003, SCOP release of March 2003, ExInt based on Genbank 122 of February 2001).

With GenePainter we developed a tool that combines protein MSA data, gene and protein structures. GenePainter maps the intron positions obtained from the gene structures to the MSA taking reading frames into account. Additionally, conserved intron positions can be displayed in provided protein structures. The output can be used to compare gene structures from the exon/intron level down to the nucleotide sequences and to resolve and improve potentially ambiguous regions in the MSAs. GenePainter does not require any additional software/database to be installed and is unique compared to previous tools in its output options and the possible application in small- as well as larges-scale analyses.

## Implementation

GenePainter was written in Ruby, does not require any additional library, and can be used on any operating system (Additional file 1). As input, GenePainter requires a protein MSA in FASTA format and corresponding gene structures in YAML format, which can be obtained for example from Scipio [17] or via the WebScipio interface [18,19]. User-specified options control the part of the alignment used in the comparison and the type of output. In addition, a PDB file can be provided to map gene structure conservation onto a protein structure. GenePainter starts with comparing sequence and gene structure file names. After processing the gene structure files, intron positions including phase information (phase 0, 1 or 2 depending on the intron’s position relative to the reading frame) and intron sequences are mapped to each sequence in the MSA. Introns are then grouped into clusters based on identical alignment positions and matched to each other. As matched introns are aligned, exons are filled with the respective placeholder for the text-based output. In the graphical output, exons are represented with their respective length, but stretched by placeholders if needed.

The text and SVG output can be processed with any appropriate software. The output to visualize intron positions mapped to structures is scripts for PyMOL [20]. The software as well as a comprehensive documentation can be found at http://www.motorprotein.de/genepainter.html.

### Needleman-Wunsch

The mapping of intron positions and phases onto a PDB file is based on an alignment of the PDB sequence with one of the sequences from the protein MSA as reference. Thus, both the reference sequence and the chain of interest from the PDB file need to be specified. The alignment is calculated as described in [21]. By default, gaps at the end of the alignment are not penalized. This adaptation is of particular importance, as reference and protein sequence may vary greatly in length, possibly leading to an inappropriate alignment. Reasons for length differences can be full-length sequence in the alignment versus sequence of a single domain in the crystal structure, protein sequence in the alignment versus sequence joined to an expression/purification tag in the structure, and missing parts in the structure due to missing electron density.

## Results and discussion

**Figure 1:**
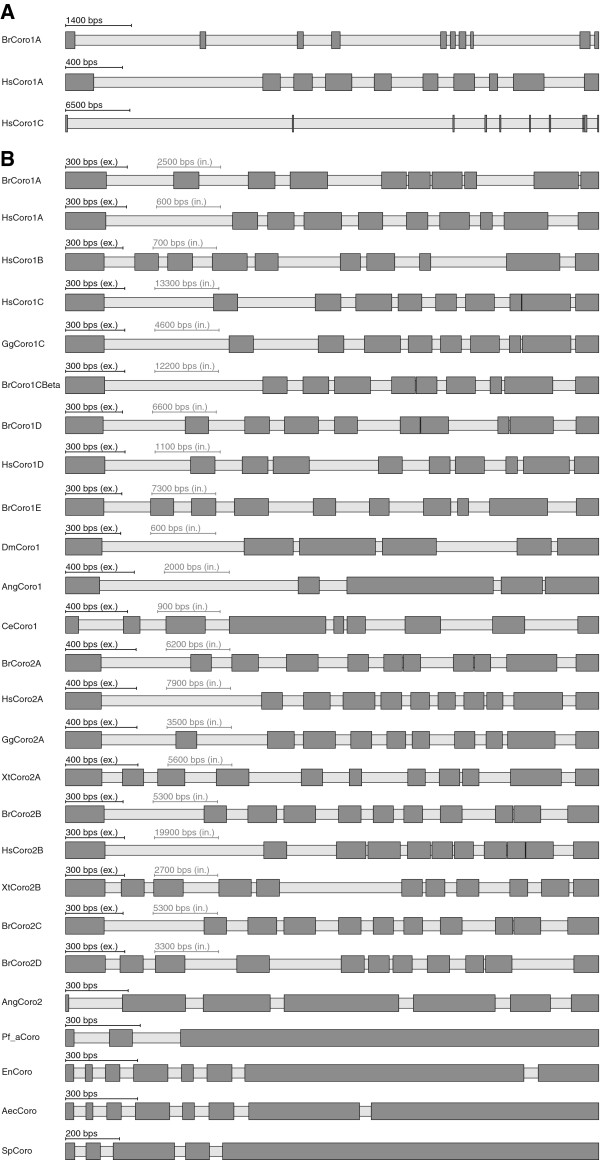
**Gene structure schemes of coronins. A**) The schemes illustrate examples of coronin genes from human (Hs = *Homo sapiens*) and zebrafish (Br = *Brachydanio rerio*) Dark grey bars and light grey bars mark exons and introns, respectively. **B**) This figure illustrates examples of coronin genes from vertebrates (Hs = *Homo sapiens*, Br = *Brachydanio rerio*, Gg = *Gallus gallus*, Xt = *Xenopus tropicalis*), arthropods (Dm = *Drosophila melanogaster*, Ang = *Anopheles gambiae*), nematods (Ce = *Caenorhabditis elegans*) and the protozoan parasite *Plasmodium falciparum* (Pf_a). In order not to make small exons vanish when very large intronic stretches are present, the scaling of introns and exons is automatically balanced to make the picture visually meaningful (scale bars for exons and introns are given, respectively). Here, except for AngCoro2 and Pf_aCoro in all schemes the introns were scaled down and the exons scaled up so that the average length of the introns equals the average length of the exons.

To demonstrate GenePainter we use part of the coronin dataset published recently [22]. For test and evaluation purposes GenePainter has also been applied to other protein families with different numbers of genes, introns per gene, and MSA lengths (Additional file 2). Coronins are a family of actin remodelling proteins consisting of a conserved β-propeller domain, that comprises the N-terminal two thirds of the sequences, a unique region, which is only conserved within closely related species, and a short C-terminal coiled-coil region that mediates trimerization. Also, a protein structure is available comprising the β-propeller domain and part of the unique region [23]. GenePainter needs FASTA formatted protein MSAs and gene structure information stored in YAML files as input (Figure 1, Additional file 1). Optionally, a protein structure can be provided in PDB format. The gene structures can most easily be obtained by using the WebScipio [18,19] web interface or via the WebScipio web service by using the provided gene_scan.rb script. The latter option requires the user to specify species names and genome assemblies, which are easier to select via the WebScipio web interface. The advantage of using Webscipio is its ability to predict protein sequences and reconstruct gene structures in cross-species searches [19] and thus the possibility to easily extend the input data by adding genes from related species. Also, Webscipio can cope with genome assembly problems like assembly gaps and sequencing errors leading to frame shifts and in-frame stop codons in exons. In the current implementation, other file formats describing gene structures like GFF [24] cannot be used as alternative input files for GenePainter. This is due to the fact that GFF files normally do not contain DNA sequence and therefore do not provide all necessary information. Optionally, alignment limits can be defined in GenePainter. This is particularly useful when comparing specific regions and domains of multi-domain proteins separately.

Because GenePainter compares gene structures based on multiple sequence alignments of proteins it can be used to analyse proteins of any degree of similarity. The coronins from the sample data comprise sequences from apicomplexans, fungi and mammals. Accordingly, the similarity of the gene structures is not obvious at first glance (Figure 1A). By scaling the exons and introns the similarity of exon lengths between homologs of closely related organisms becomes suggestive (Figure 1B. Note the different scaling of exons and introns in this figure). Exons and introns were scaled up and down, respectively, so that the average length of the exons equals the average length of the introns. GenePainter maps intron positions including phase to the sequences as provided in the multiple sequence alignments.

### Gene structure alignments

**Figure 2:**
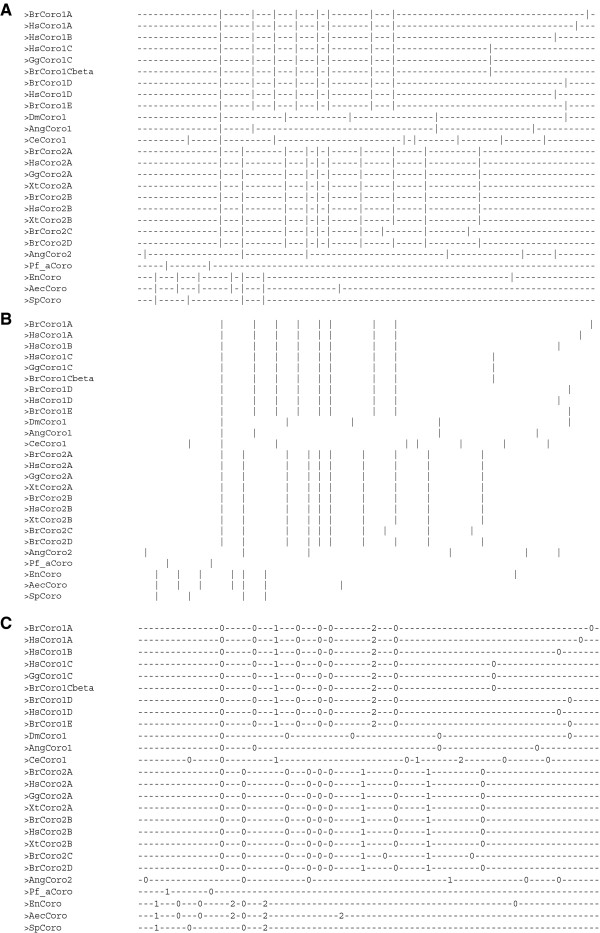
**Gene structure alignments.** The gene structures shown in Figure 1 were aligned with GenePainter. Three visualization options focusing on common introns exist. In each, exons and introns are represented independent of their length. **A**) In this gene structure alignment, coding sequences are represented by “-“ and introns by “|”. “|” underneath each other indicate intron positions with the same phase at the same position in the multiple sequence alignment. **B**) Here, only introns are pictured. Coding sequences are denoted by spaces, introns by “|”. **C**) Similar visualization as in **A**) except that the intron phases are given instead of the intron indicator “|”. All outputs shown are plain text files and can be analysed with any text editor.

**Figure 3:**
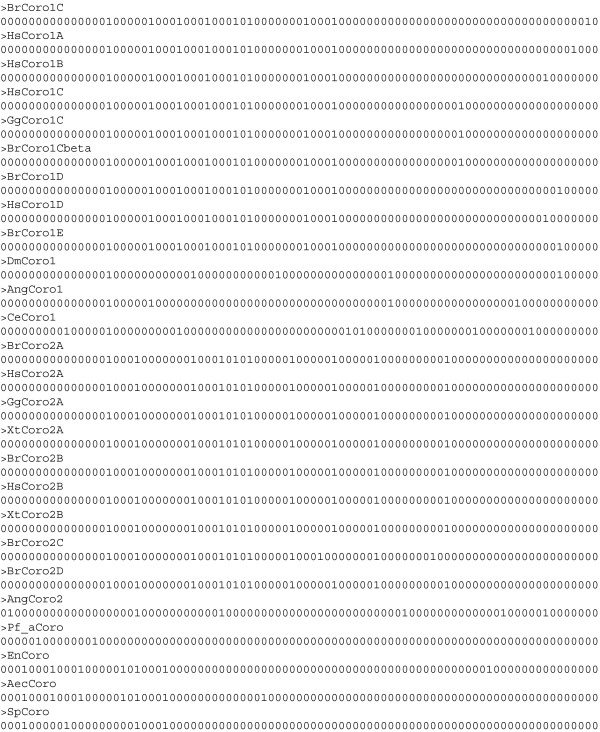
**Binary representation of gene structures.** The binary representation of aligned intron positions is in FASTA format. The presence and absence of common introns are denoted by ones and zeros, respectively.

The aligned gene structures can be analysed in various formats. The basic output format displays common intron positions in plain text, where introns are represented by vertical bars “|“ (Figure 2A). Hyphen-minuses “-“ are used as spacers for better orientation and represent exonic sequence in abstract form. Optionally, the hyphen-minuses can be replaced by spaces so that the output just represents common introns (Figure 2B). These formats are particularly useful for visualizing large-scale data (many positions and sequences in the MSAs) and are independent of exon and intron lengths. Additional information can be added by using the -n option of GenePainter by which intron positions are represented by phase numbers instead of vertical bars “|“ (Figure 2C). With the option –phylo the common intron data is transformed into an intron present “1” – intron absent “0” format, which can be used to calculate phylogenetic trees based on gene structure data (Figure 3).

**Figure 4:**
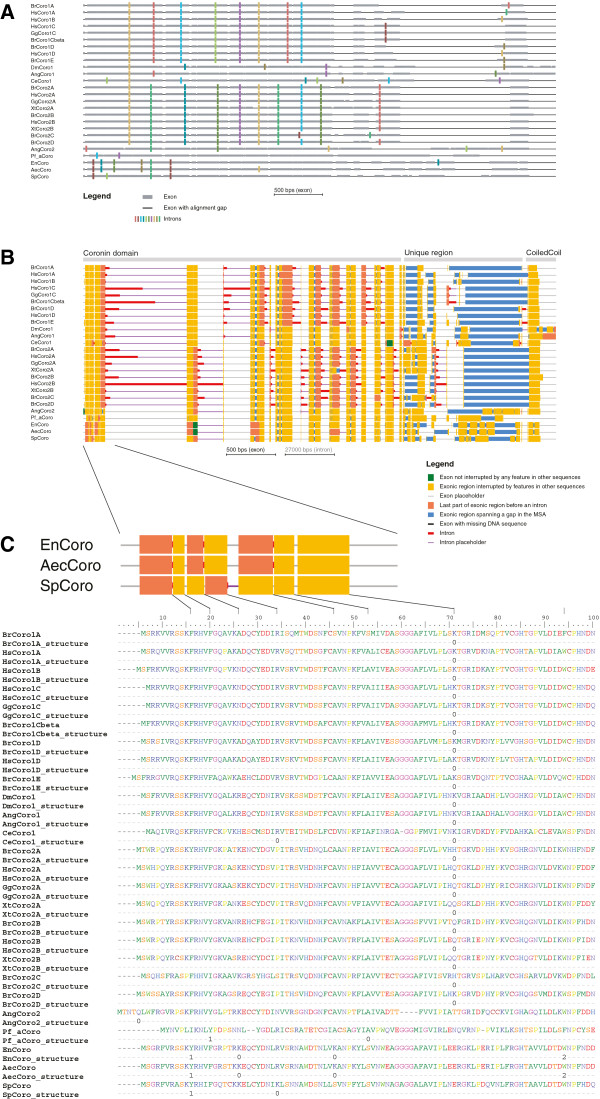
**Nucleotide level alignment of gene structures. A**) Representation of gene structures with aligned introns. Grey bars represent exons and coloured lines introns. Exons are drawn to scale, while introns are represented in a length independent way. Introns at the same position are drawn in the same colour. The output also accounts for alignment gaps, which are drawn with thin black lines. **B**) Representation of the gene structures at the nucleotide level. Exons and introns are scaled that both represent 50% of the width of the figure. Without scaling, the introns would dominate the schemes. Red and magenta lines represent introns and intron gaps, respectively. Intron gaps are placeholders to fill the space of shorter introns compared to the longest intron at that position up to the next exon. The thick bars denote sequence within exons (green, orange and coral bars) and gap positions within exons (smaller blue bars) that were inserted into the protein sequences to adjust the multiple sequence alignment. Different colours for exonic sequences have been introduced to emphasize particular aspects like exons, which are not interrupted by sequence alignment gaps or introns in any of the other sequences (green bars) and the last uninterrupted parts of exonic sequence before introns (orange bars). The last option is particularly useful to identify the ends of exonic sequence before very short introns, or to identify introns in very huge alignments. Coral bars denote all other exonic sequence. Light gray lines symbolize placeholders within exonic sequences that are interrupted by introns in other sequences. All placeholders and markers for alignment gaps are added to optically align the corresponding exonic sequences beneath each other. **C**) Section of the gene structure alignment of B) with respect to the multiple sequence alignment to highlight the exon and intron features.

A gene structure alignment including exon and intron lengths is shown in Figure 4. The same alignment is drawn with varying degrees of detail (Figure 4A, 4B). In the most reduced picture the focus lies on common introns (Figure 4A). The intron length is not included in this figure, but in Figure 4B, which provides most information about the underlying gene structures. Here, it becomes immediately obvious that intron lengths vary considerably (compare human *Hs*Coro2B and frog *Xt*Coro2B for example). In addition, the scheme shows that the N-terminal β-propeller domain of coronin is highly conserved while the unique regions are variable and contain many gaps in the multiple sequence alignment (blue bars; Figure 4B).

The gene structure information can also be incorporated into the protein MSA as additional lines (option -a) where intron positions are either displayed as vertical bars “|“ or as numbers defining the phase of the respective introns (Figure 4C). This format is most useful if the MSA will be re-evaluated to identify miss-aligned positions and regions.

### Visualizing conserved intron positions on protein structures

**Figure 5:**
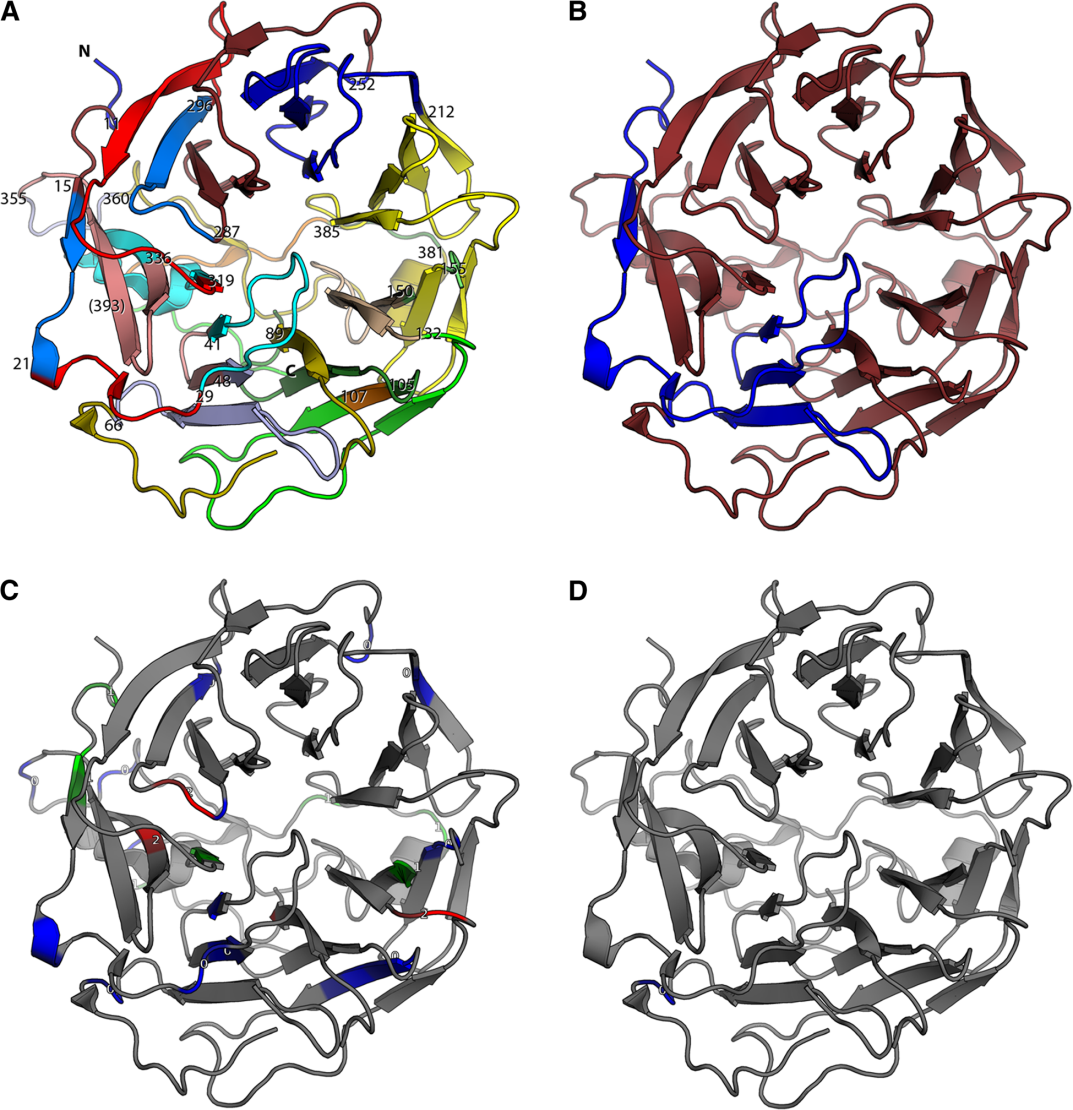
**Visualization of gene structures on protein structures.** In this figure, intron positions and phases are visualized in a protein structure. The gene structure of human *Hs*Coro1A (see Figure 1) was mapped onto the Coro1A structure from mouse [23]. The two different output formats are shown for comparison. **A**) Illustrates colouring of exons mapped to the protein structure (color_exons.py). For better orientation, the N- and C-termini and the positions of the last residues in each putative exon are given. The number in brackets denotes an intron position covered by another structural element. **C**) Displays the phases of the introns (color_splicesites.py). Numbers indicate the respective intron phases. In both figures, introns occurring in any of the sequences within the MSA are shown (-consensus 0). Contrary, introns conserved in 80% of all proteins (default value) are shown in **B**) and **D**). Analogous to **A**) and **C**), **B**) refers to color_exons.py and **D**) to color_splicesites.py.

Gene structure conservation derived by GenePainter can further be mapped on protein structures (option –pdb <file> [chain]). Therefore, one of the proteins from the MSA (set by -pdb_prot) is taken as reference and aligned with the protein sequence from a PDB file. Generating the alignment with standard Needleman-Wunsch parameters, which penalize gaps at the end of the alignment, can be forced by setting the option -penalize_endgaps. Based on this alignment, intron positions and phases are projected onto the protein structure. If reference gene and protein structure chain are not specified, the first sequence in the alignment and chain A will be used by default. GenePainter generates two python scripts for execution within PyMol comprising all necessary steps (including loading the PDB) in order to display the mapped intron positions and phases. While the script color_exons.py colours residues based on the underlying gene structure (Figure 5A), the other highlights only intron phases (color_splicesites.py; Figure 5C). In this visualization, both the last and first residues of succeeding exons are coloured by a three-colour scheme denoting the phases of the respective introns. In order to elucidate the conservation of the respective intron positions, by default only those positions that are conserved in more than 80% of the genes of the alignment (parameter can be changed via -consensus) are considered for visualization (Figures 5B and 5D). In both visualizations attention is focused on those parts of the structure, on which intron data are mapped. Unused chains and regions not mapped to the reference sequence like cloning artefacts and protein purification tags are displayed in grey.

### Limits and possible applications of GenePainter

In the current version, GenePainter is limited to group introns to common introns only if these appear at the exact same position and exact same reading frame in the aligned protein sequences. In contrast CIWOG defines introns, which happen in closely neighbouring amino acids, as common introns [11]. However, there are many examples of very short exons not only in mammals but also in other branches of the eukaryotes, which would unnecessarily be grouped as common introns. As we showed (Figure 4C) more and more conserved introns will appear in the alignment as soon as more sequences and gene structures are added. For instance, the *Schizosaccharomyces pombe* (*Sp*Coro) and *Caenorhabditis elegans* (*Ce*Coro1) coronin genes share an intron at the N-terminus (Figure 4C). We believe that almost all introns are shared between at least two genes from two different species. However, most analyses do not cover enough data, not enough sequences and not enough species. We have shown previously that all introns within the arthropod muscle myosin heavy chain genes are shared between at least two species, which became only obvious after having analysed genes from 22 species [9]. It is highly unlikely that introns would have been introduced at the exact same position with exact same reading frame independently in species whose last common ancester had been millions of years ago (600 myr in case of the arthropods). Data from further arthropods showed, that introns initially found to be shared by only two species are actually shared by more species, and that the last common ancestor of the arthropods must have had even more introns than assumed before (data available from CyMoBase [25]). If data from more species and more protein families would be included, shared introns in genes present in the last common ancestor of the eukaryotes could be identified. For other applications that do not demand strict intron conservation, GenePainter could be extended to allow variable positional flexibility.

Binary data can be very useful to reconstruct phylogenetic relationships and is often created from morphological data [26-28]. We used binary data obtained from protein family inventories (subfamily homolog present or absent) to reconstruct the phylogeny of 21 arthropod species [29]. The resulting phylogeny was in agreement with that derived from protein sequence data. The intron pattern of a protein family (Figure 3) can therefore also be used for phylogenetic tree reconstructions, or for combined data analyses. Depending on the taxonomic distribution of the included sequences, intron pattern data of several protein families need to be included to derive discriminative and meaningful information.

## Conclusions

GenePainter is a tool to analyse the conservation of gene structures of eukaryotic proteins. It aligns the gene structures to the respective protein sequences in a multiple sequence alignment. Gene structure conservation can be displayed in a binary format (exons and introns) and based on the nucleotide sequences. GenePainter can map gene structure conservation on protein structures and provides scripts for visualization in PyMol. Therefore, GenePainter will be a valuable tool for gene structure guided improvements of multiple sequence alignments and for phylogenetic analyses including or focusing on the conservation of intron positions within eukaryotic genes.

## Availability and requirements

**Project name:** GenePainter

**Project home page:**http://www.motorprotein.de/genepainter.html

**Operating system:** Platform independent

**Programming languages:** Ruby

**Software requirements:** Ruby version 1.9.2 or higher

**License:** GenePainter can be downloaded and used under a GNU General Public License.

**Any restrictions to use by non-academics:** Using GenePainter by non-academics requires permission.

## Competing interests

The authors declare that they have no competing interests.

## Authors’ contributions

MK, FO, and SW designed the project and set the requirements of the tool. BH and FO wrote the source code. SM added the mapping of the gene structure patterns to PDB files. MK, BH and SM wrote the manuscript. All authors read and approved the final manuscript.

## Supplementary Material

Additional file 1 **This file contains the GenePainter software **(gene_painter.rb), **a README file for installation instructions, the GenePainter documentation, and a script to reconstruct genes via the web service of WebScipio **(gene_scan.rb). It also includes example data (MSA, gene and protein structures) used to create the figures. This file is also available from the project homepage. Click here for file

Additional file 2 **This file contains a benchmark test of GenePainter on coronin (552 sequences, 1144 alignment positions), dynactin1 (207 sequences, 2112 alignment positions), dynactin3 (213 sequences, 428 alignment positions), Wiskott Aldrich Syndrome protein (229 sequences, 2051 alignment positions), and myosin heavy chain genes (2640 sequences, 9214 alignment positions).** All sequences and gene structures can be found at CyMoBase (//www.cymobase.org). Click here for file
