# Supplementary material for: GenePainter: a fast tool for aligning gene structures of eukaryotic protein families, visualizing the alignments and mapping gene structures onto protein structures
Source: BMC Bioinformatics. 2013 Mar 4;14:77. doi: 10.1186/1471-2105-14-77 (PMC3605371; doi:10.1186/1471-2105-14-77)
Supplement: Additional file 1 — This file contains the GenePainter software (gene_painter.rb), a README file for installation instructions, the GenePainter documentation, and a script to reconstruct genes via the web service of WebScipio (gene_scan.rb). It also includes example data (MSA, gene and protein structures) used to create the figures. This file is also available from the project homepage. [file 1471-2105-14-77-S1.zip › gene_painter/documentation.pdf]

GenePainter - Documentation

Authors

Björn Hammesfahr, Florian Odronitz, Stefanie Mühlhausen, Stephan Waack, Martin Kollmar

How to cite

GenePainter: a fast tool for aligning gene structures of eukaryotic protein families, visualizing the alignments and mapping gene structures onto protein structures, Submitted to BMC Bioinformatics

Contact

Dr. Martin Kollmar
Group Systems Biology of Motor Proteins
Department of NMR-based Structural Biology
Max-Planck-Institute for Biophysical Chemistry
Am Faßberg 11
37077 Göttingen

mako@nmr.mpibpc.mpg.de

Homepage

http://www.motorprotein.de/genepainter.html

Licence

Licence: GenePainter can be downloaded and used under a GNU General Public License.
Any restrictions to use by non-academics: Using GenePainter by non-academics requires permission.

Table of contents

Authors ... 1
How to cite ... 1
Contact ... 1
Homepage ... 1

Licence ... 1

Table of contents ... 2

Introduction ... 3

Installation ... 3
Unpack ... 3
Compilation ... 3
Ruby version ... 3

Usage ... 3
Ruby interpreter ... 4

Example ... 4

Options ... 4

Input ... 5
Multiple protein sequence alignment ... 5
YAML ... 6

Meaning of the parameters ... 8
Text-based output ... 8
Graphical output ... 10
Gene structures mapped to protein structures ... 12

Introduction

The conservation of intron positions comprises information useful for de novo gene prediction, protein sequence alignment improvement, and for analyzing the origin of introns. Here, we present GenePainter, a standalone tool for mapping gene structures onto protein multiple sequence alignments (MSA). Gene structures, as obtained for example by using WebScipio (http://www.webscipio.org), are aligned with respect to the exact positions of the introns (down to nucleotide level) and intron phase. The output can be visualized in various formats, ranging from plain text to complex graphical figures.

Installation

Unpack

Use one of the following methods, depending on the archive file type:

```
$ unzip gene_painter.zip
$ tar -xzf gene_painter.tgz
```

Compilation

No compilation required.

Ruby version

Ruby version 1.9.2 or higher is required. If necessary, consider using Ruby Version Manager (https://rvm.io/; RVM) to install and work with multiple ruby environments on your machine.

Usage

```
$ ruby gene_painter.rb -i <alignment> -p <yaml-files> \
[<options>]
```

**Option**	Description
**-i**	Multiple protein sequence alignment in FASTA format.
**-p**	Path to the directory containing the gene structures in YAML-format.

A more detailed description of the MSA and the YAML files as well as the incorporation of both by GenePainter is given in the Input section.

Ruby interpreter

Invoke GenePainter via one of the following options:

1. As a script
   `$ ruby gene_painter.rb`
2. As a program
   `$ ./gene_painter.rb`

Important note

GenePainter assumes your ruby interpreter to be located at `/usr/local/bin/ruby`. This assumption is coded in the first line of `gene_painter.rb` (Shebang). To meet your local requirements, please edit the Shebang and change the specified path to the correct one. With the Ruby interpreter located at `/usr/bin/ruby`, the Shebang should look like this: `#!/usr/bin/ruby`.

Example

```
$ ruby gene_painter.rb \
-i test_data/coronin_alignment.fas  -o coronin \
-p test_data/coronin_genes -svg 1000 500 extended \
-pdb test_data/2AQ5.pdb -pdb_prot HsCoro1A
```

Options

Option	Description
**-o <project_name>**	Base name of the output file(s) (default: 'genepainter').
**-a**	Output the alignment file with additional lines containing intron phases.
**-n**	Mark introns by intron phase instead of the vertical bar "|".
**-phylo**	For phylogenetic analysis: Mark exons and introns by "0" and "1", respectively.
**-s**	Mark exons by " " instead of "-" and introns by "|".
**-svg width height [extended | normal | reduced]**	Create an SVG-file of size <width> x <height>.<br>Use this parameter to create a more detailed svg.<br>Use this parameter to create the normal svg (default).<br>Use this parameter to create a svg focused on common introns.

Option	Description
**-start X**	Alignment position to start (default: position 1).
**-stop Y**	Alignment position to stop (default: last position).
**-pdb file.pdb [chain]**	Two scripts for execution in PyMol will be provided. In color_exons.py the consensus exons are colored and in color_splicesites.py the splice junctions of the consensus exons are marked for <chain> (default: chain A).
**-pdb_prot prot_name**	Use protein <prot_name> as reference for alignment with the pdb sequence Default: First protein in <alignment>.
**-f**	Force alignment between pdb and first protein sequence of the MSA or protein <prot_name> (if specified). This ignores the default that intron positions will only be mapped if the alignment score > 70%.
**-consensus value**	Color only intron positions conserved in <value> percent of all genes (default: 80%).
**-ref_prot_struct**	Color only the intron positions occurring in the gene of the reference protein. May be combined with "-consensus".
**-penalize_endgaps**	Penalize gaps at the end of the alignment (behaves like the standard Needleman-Wunsch algorithm). Default: gaps at the end of the alignment are not penalized.

Input

GenePainter expects two types of input:

1. a FASTA-formatted multiple sequence alignment (MSA);
2. a folder containing gene structures in YAML format as specified by WebScipio.

GenePainter combines information from the alignment with gene structures. Therefore, the protein names from the MSA are matched with YAML filenames. Only those genes, which can be matched (i.e. protein name equals the YAML filename) will be analysed.

Multiple protein sequence alignment

This file must be a multiple protein sequence alignment, in which all sequences are of same length. Protein sequences are matched with the gene structures on the basis of the FASTA header and file names, respectively. To this end, the FASTA header must be exactly like the corresponding YAML filename for each gene, which should be included in the analysis. For this reason, FASTA header must not contain any blanks or special characters.

YAML

For the analysis, GenePainter needs gene structures information for each gene in a specific file format, the YAML-format as generated for example by WebScipio (http://www.webscipio.org) (Figure 1). Moreover, all gene structures should be located in the same directory.

The most convenient way to obtain YAML-formatted files is to use WebScipio for gene reconstruction and download the resulting YAML file. As an alternative to the web interface, one can also download the Scipio command line tool from the web interface. However, one can also rebuild YAML files according to the specifications made by WebScipio. To this end, information about available keys as well as an example file is shown below (Figures 1-4).

```
---
- matchings:
  - mismatchlist: []

    prot_start: 0
    nucl_end: 198
    prot_end: 66
    translation: MSRQVVRSSKFRHVFGQPAKADQCYEDVRVSQTTWDSGFCAVNPKFVALICEASGGGAFLVLPLGK
    dna_start: -72598366
    type: exon
    seqshifts: []

    nucl_start: 0
    seq: atgagccggcaggtggtccgctccagcaagttccgccacgtgtttggacagccggccaaggccgaccagtgctatgaag
    dna_end: -72598168
  - dna_start: -72598168
    type: intron
    nucl_start: 198
    seq: gtgagcccctggggccctgggggagcagctcctccaccggacccatggctctgtgcaggtcctgattaggtgacggtc
    dna_end: -72596978
  - mismatchlist: []

...

    prot_start: 427
    nucl_end: 1383
    prot_end: 461
    translation: DAVSRLEEEMRKLQATVQELQKRLDRLEETVQAK
    dna_start: -72594716
    type: exon
    seqshifts: []

    nucl_start: 1281
    seq: gatgccgtgtctcggctggaggaggagatgcggaagctccaggccacggtgcaggagctccagaagcgcttggacaggc
    dna_end: -72594614
  unmatched: 0
  prot_start: 0
  stopcodon: tag
  downstream: tagagccccgcagggcctccagcagggtcagccattcacacccatccactcacctcccattcccagccacatgg
  additional: 0
  prot_end: 461
  ID: 720
  dna_start: -72598366
  reason:
  downstream_gap:
  upstream_gap:
  target_len: 75226909
  strand:
  -
```

Figure 1 – Except from the YAML-file describing HsCoro1A. This file is part of the test data included in the package. For illustration purposes, all exon and intron descriptions but the very first and very last ones have been omitted (marked in yellow). Exons are highlighted by green boxes and introns by red boxes. The keys listed in the black boxes do not describe exons or introns but the BLAT hit in general.

Keys for BLAT hits

Every BLAT hit is described by the following keys:

`ID`	The id of the BLAT hit (equals the line number in the psl file).
`status`	Might be "complete", "partial", "incomplete" or "manual". "complete" means that Scipio had no problems locating the query; "partial" means that the hit is on one of multiple targets each matching a part of the query; "incomplete" means that Scipio could not completely match the query sequence to the target; another reason for "incomplete" is a missing stop-codon in the target sequence following the last amino acid of the query sequence. "manual" can be entered if the output was modified by hand.
`reason`	If `status` is "incomplete", the reason why.
`prot_len`	The length of the query (in amino acid coordinates).
`prot_start`	Start of the matched part of the query if larger than zero.
`prot_end`	End of the matched part of the query if less than `prot_len`.
`prot_seq`	The query sequence (in a partial hit: only the matched part).
`target`	The name of the target sequence.
`target_len`	The total length of the target sequence.
`strand`	"+" (= forward) or "-" (= reverse).
`dna_start`<br>`dna_end`	The location of the hit.
`matches`	The number of matches if less than `prot_len`.
`mismatches`	The number of mismatches.
`undetermined`	The number of undetermined residues.
`unmatched`	The number of unmatched query residues.
`additional`	The number of additional residues in the translated target.
`score`	The score of the hit.
`upstream`	DNA Sequence upstream of hit, ending before start codon.
`upstream_gap`	(unaligned) DNA Sequence preceding a partial hit.
`matchings`	The locations of exons and introns, see next section.
`stopcodon`	Stop codon if present.
`downstream`	DNA Sequence downstream of hit, starting with stop codon if present.
`downstream_gap`	(unaligned) DNA Sequence following a partial hit.

The commandline parameter `--show = `*<comma-separated list>* can be used to choose a user-defined collection of keys to be shown.

For the alignment of the query to the translation, `unmatched` counts the gap characters inserted into the translation (= residues present only in the query), `additional` counts gap characters inserted into the query (= residues present only in the translation), `matches+mismatches+undetermined` counts residues present in both (see also comments to `seqshifts` below).

Figure 2 - Overview about all keys WebScipio uses to generate the YAML-output.

Keys for matchings

Every matching (intron/exon) is given with the following keys:

`type`	"intron", "intron?", "exon", or "gap". "intron?" is used for uncertain introns (no regular splice patterns found)
`nucl_start`<br>`nucl_end`	Location in the query (in nucleotide coordinates; `nucl_end` not for introns).
`dna_start`<br>`dna_end`	Location in the target.
`seq`	DNA sequence of the feature.

Figure 3 – Overview about keys WebScipio uses to describe introns and exons.

Keys that appear only in exons:

seqshifts	A list of locations of sequence shifts. Each entry of the list consists in turn of four keys (nucl_start/end, dna_start/end). If nucl_start = nucl_end, the target sequence between dna_start < dna_end is translated and counted as additional residue(s); if dna_end - dna_start is not a multiple of 3, the extra single nucleotides causing the frameshift are translated as 'X'. If nucl_start < nucl_end and dna_start = dna_end, the corresponding part of the query sequence is missing in the translation and counted as unmatched. A frameshift caused my missing nucleotides is represented by a target segment of length 1 or 2 corresponding to one query residue (counted as undetermined). nucl_end - nucl_start is always a multiple of 3.
mismatchlist	Positions of mismatches (in amino acid coordinates).
undeterminedlist	Positions of undetermined residues (in query coordinates).
inframe_stopcodons	Positions of in-frame stopcodons (in query coordinates).
translation	Translation of the aligned part of the DNA sequence.
overlap	Number of nucleotides at the end of the target that are identical with the beginning of the following target, and not considered part of this location.

The commandline parameters --show_exon = <...>, --show_intron = <...>, --show_gap = <...> can be used to choose user-defined output for matchings. The following additional keys can be activated by this:

nucl_pos	In introns, a synonym for nucl_start(=nucl_end)
prot_start	The location transformed into residue coordinates rather than nucleotides. A remainder of 1 is rounded down, and 2 is rounded up.
prot_end	
prot_pos	(this one for introns)
prot_seq	Part of the query matching an exon, or unmatched part in a gap.

Figure 4 – Overview about all keys WebScipio uses only for exons.

Meaning of the parameters

The following figures illustrate some of GenePainters output formats and options. All figures were generated with test data comprising coronin genes as included in the archive `gene_painter.zip`.

Text-based output

The basic output format is a plain text-file where exons are represented as minus signs and introns as vertical bars (Figure 5A). A more detailed output including intron phases can be obtained by using the `-n` option (Figure 5C). By using the `-s` option (Figure 5B), only introns are represented by "|". Moreover, intron phases can be included as additional lines in the given alignment (Figure 6A; option `-a`), or an alignment based on the presence 1 and absence 0 of introns for further phylogenetic analyses can be generated (Figure 6B; option `–phylo`).

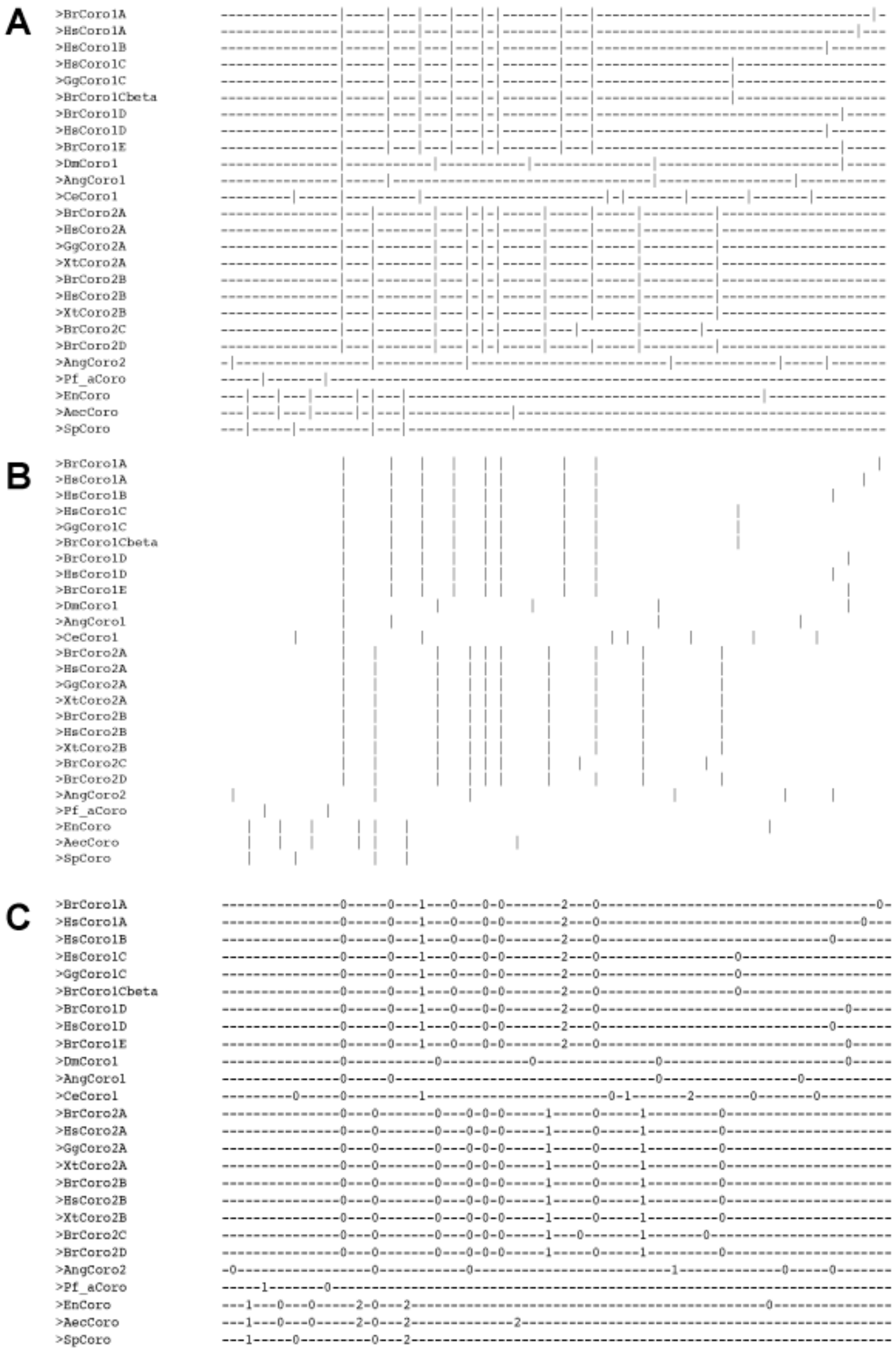

Figure 5 - Basic output formats of GenePainter.

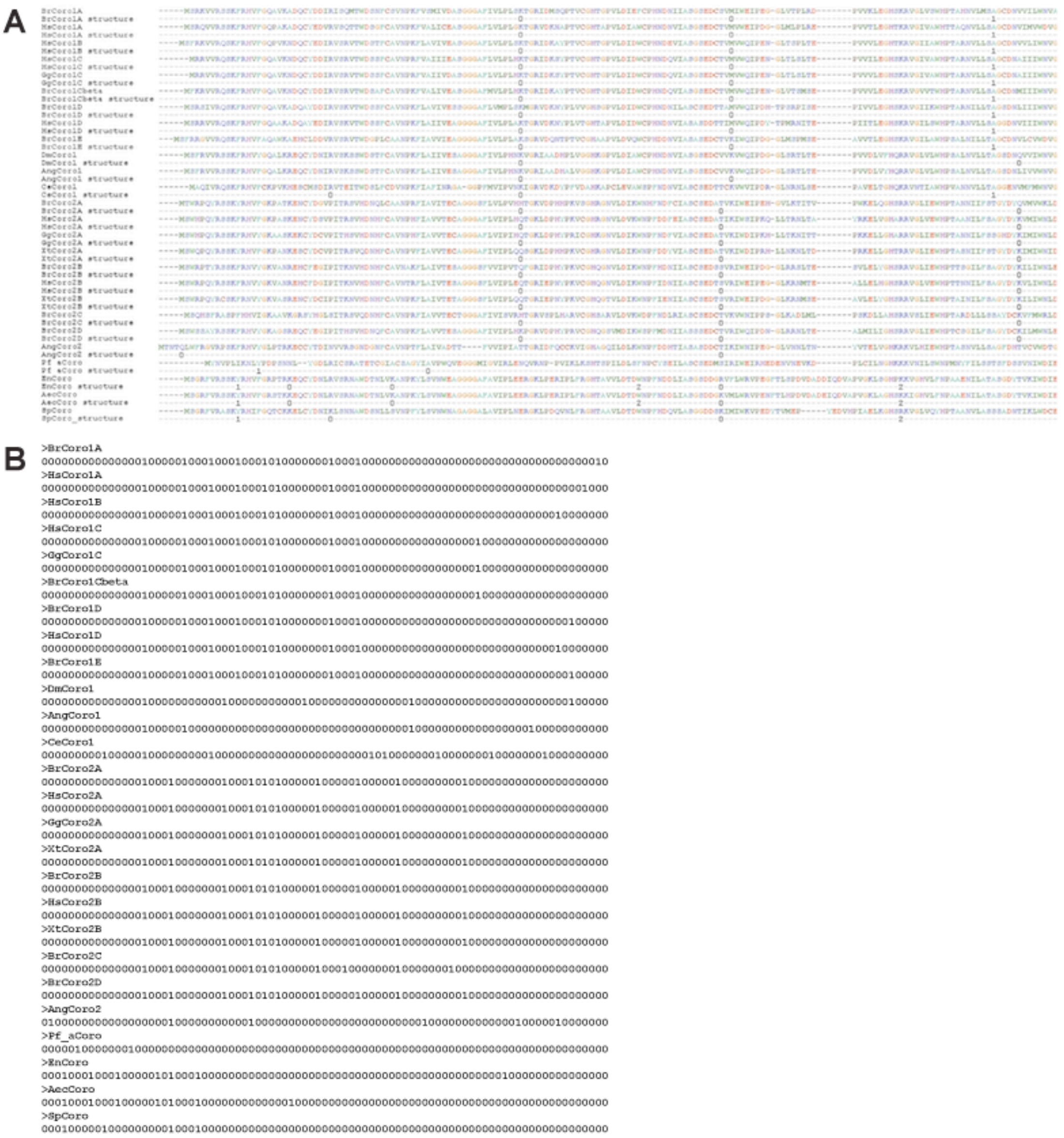

Figure 6 - Intron phases included in given alignment.

Graphical output

Apart from text based output formats, a graphical output can be generated. To this end, SVG parameters need to be set with `-svg <width> <height> [extended|normal|reduced]`. Figure 7A illustrates a detailed SVG of size 1000 x 500 pixel (`extended` is set). In contrast, the basic SVG (same size, but with `normal` set) is shown in Figure 7B. In Figure 7C the `reduced` SVG is shown, which highlights common introns. In order to restrict the algorithm to a certain part of the alignment, first and last positions to be considered can be set by `-start` and `-stop`, respectively. Figure 8 pictures a detailed SVG based on only part of the given alignment (domain of interest, alignment positions `1-612`).

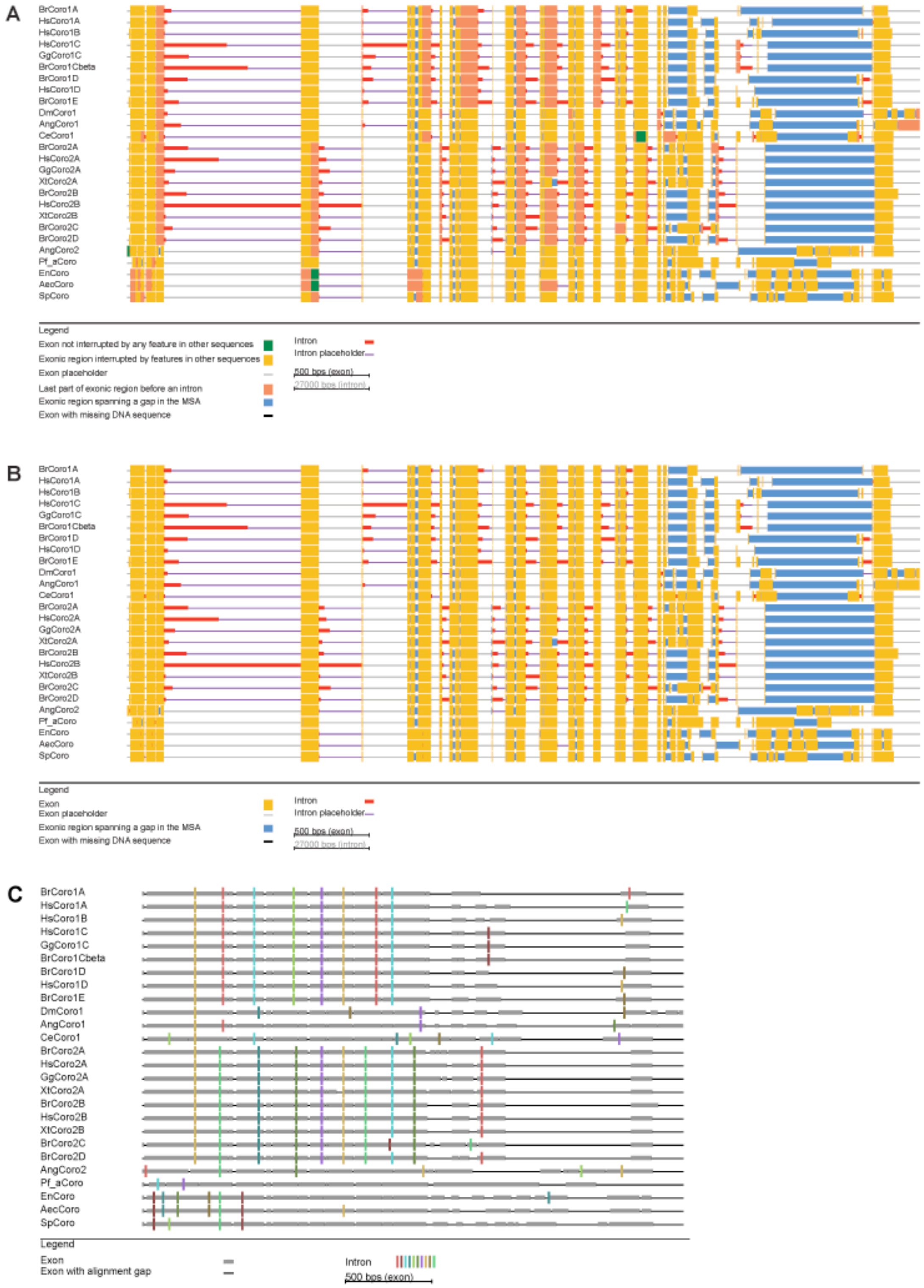

Figure 7 - Graphical output of GenePainter.

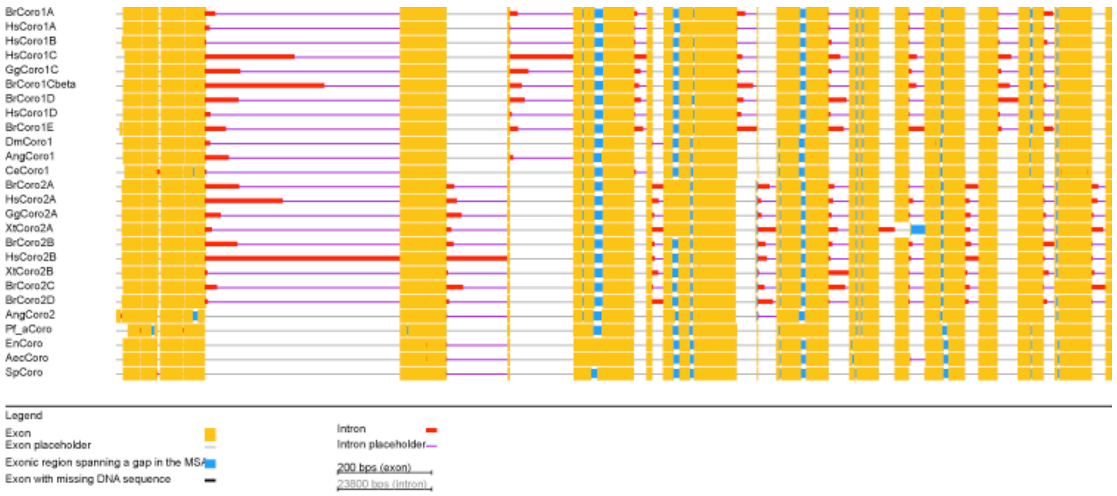

Figure 8 - Detailed graphical output covering only domain of interest.

Gene structures mapped to protein structures

Additionally, if a pdb file is specified via `-pdb`, intron positions and phases are mapped onto protein structure. Figure 9A demonstrates mapping of the exons of the human coronin HsCoro1A gene (`-pdb_prot HsCoro1A`) onto the protein structure of mouse coronin MmCoro1A (the pdb file is part of the test data set, `-pdb test_data/2Aq5.pdb`). While for this figure all exons that are conserved in at least 80% of all proteins are considered (default), Figure 9B displays all exons present in the reference sequence (`-ref_prot_struct`). Accordingly, splice sites are shown in Figures 9C and 9D. In this output, attention is drawn to intron phases. A three-color scheme and numbers denote phases.

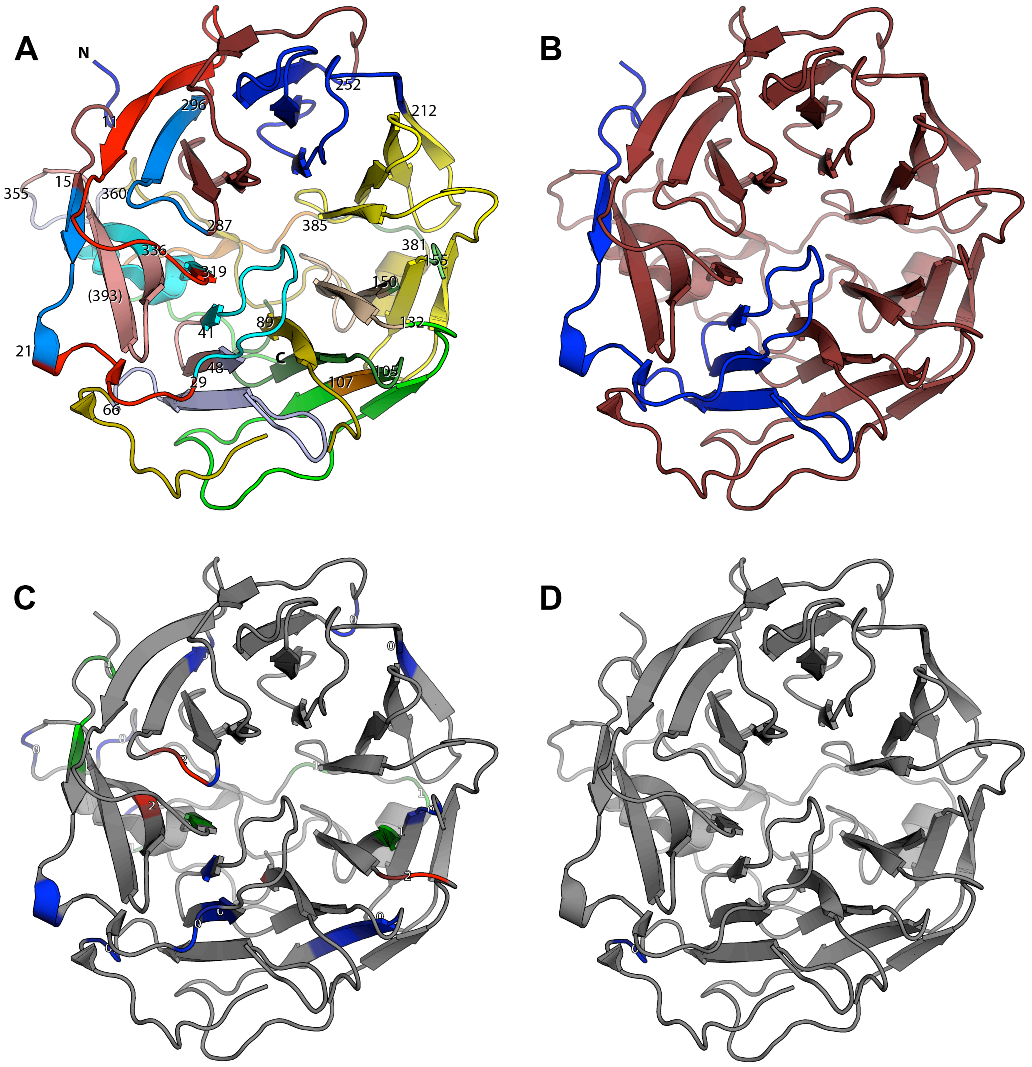

Figure 9 - Mapping of conserved exons (9A, 9B) and intron position and phase (9C, 9D) onto protein structure.

Part of the underlying algorithm is the calculation of a global alignment between reference and pdb sequence. Although this alignment is a simple implementation of the Needleman-Wunsch algorithm, some adjustments are done. In detail, gaps at the end of the alignment are not penalized. This is particular useful, as pdb sequence and reference sequence may vary in length. Alignments with and without free end gaps are opposed in Figure 10.

```
HsCoro1A:
MSRQVVRSSKFRHVFGQPAKADQCYEDVRVSQTTWDSGFCAVNPKFVALICEASGGGAFLVLPLGKTGRVDKNAPTVCGHTAPVLDIAWCPHNDNVIASGSEDCTVMVWEIPDGGLM
LPLREPVVTLEGHTKRVGIVAWHTTAQNVLLSAGCDNVIMVWDVGTGAAMLTLGPEVHPDTIYSVDWSRDGGLICTSCRDKRVRIIEPRKGTVVAEKDRPHEGTRPVRAVFVSEGKI
LTTGFSRMSERQVALWDTKHLEEPLSLQELDTSSGVLLPFFDPDTNIVYLCGKGDSSIRYFEITSEAPFLHYLSMFSSKESQRGMGYMPKRGLEVNKCEIARFYKLHERRCEPIAMT
VPRKSDLFQEDLYPPTAGPDPALTAEEWLGGRDAGPLLISLKDGYVPPKSRELRVNRGLDTGRRRAAPEASGTPSSDAVSRLEEEMRKLQATVQELQKRLDRLEETVQAK

2AQ5.pdb, global end gap free alignment
-------SSKFRHVFGQPAKADQCYEDVRVSQTTWDSGFCAVNPKFMALI-EASGGGAFLVLPLGKTGRVDKNVPLV-GHTAPVLDIAW-PHNDNVIASGSEDCTVMVWEIPDGGLV
LPLREPVITLEGHTKRVGIVAWHPTAQNVLLSAG-DNVILVWDVGTGAAVLTLGPDVHPDTIYSVDWSRDGALICTSCRDKRVRVIEPRKGTVVAEKDRPHEGTRPVHAVFVSEGKI
LTTGFSRMSERQVALWDTKHLEEPLSLQELDTSSGVLLPFFDPDTNIVYLCGKGDSSIRYFEITSEAPFLHYLSMFSSKESQRGMGYMPKRGLEVNK-EIARFYKLHERKCEPIAMT
VPRKSDLFQEDLYPPTAGPDPALTAEEWLGGRDAGPLLISLKDGYVPPKSR-----------------------------------------------------------

2AQ5.pdb, global alignement without end gap free option
-------SSKFRHVFGQPAKADQCYEDVRVSQTTWDSGFCAVNPKFMALI-EASGGGAFLVLPLGKTGRVDKNVPLV-GHTAPVLDIAW-PHNDNVIASGSEDCTVMVWEIPDGGLV
LPLREPVITLEGHTKRVGIVAWHPTAQNVLLSAG-DNVILVWDVGTGAAVLTLGPDVHPDTIYSVDWSRDGALICTSCRDKRVRVIEPRKGTVVAEKDRPHEGTRPVHAVFVSEGKI
LTTGFSRMSERQVALWDTKHLEEPLSLQELDTSSGVLLPFFDPDTNIVYLCGKGDSSIRYFEITSEAPFLHYLSMFSSKESQRGMGYMPKRGLEVNK-EIARFYKLHERKCEPIAMT
VPRKSDLFQEDLYPPTAGPDPALTAEEWLGGRDAGPLLISLKDGYVPPK------------------------------S---------------------R--------
```

Figure 10 - Aligned sequences.
