# Supplementary material for: GenePainter: a fast tool for aligning gene structures of eukaryotic protein families, visualizing the alignments and mapping gene structures onto protein structures
Source: BMC Bioinformatics. 2013 Mar 4;14:77. doi: 10.1186/1471-2105-14-77 (PMC3605371; doi:10.1186/1471-2105-14-77)
Supplement: Additional file 2 — This file contains a benchmark test of GenePainter on coronin (552 sequences, 1144 alignment positions), dynactin1 (207 sequences, 2112 alignment positions), dynactin3 (213 sequences, 428 alignment positions), Wiskott Aldrich Syndrome protein (229 sequences, 2051 alignment positions), and myosin heavy chain genes (2640 sequences, 9214 alignment positions). All sequences and gene structures can be found at CyMoBase (//www.cymobase.org). [file 1471-2105-14-77-S2.pdf]

Additional file 2

This file contains a benchmark test of GenePainter on coronin, dynactin1, dynactin3, Wiskott Aldrich Syndrome protein (WASP), and myosin heavy chain genes. All sequences and gene structures can be found at CyMoBase (http://www.cymobase.org).

Protein family	# Sequences	Alignment length (positions)	Benchmark (seconds)
Coronin	552	1144	65.492
Dynactin1	207	2112	22.480
Dynactin3	213	428	1.971
WASP	229	2051	9.124
Myosin heavy chain	2640	9214	9,519.729
